# Clinical characteristics and prognostic factors of bone lymphomas: focus on the clinical significance of multifocal bone involvement by primary bone large B-cell lymphomas

**DOI:** 10.1186/1471-2407-14-900

**Published:** 2014-12-02

**Authors:** Huanwen Wu, Marilyn M Bui, Douglas G Leston, Haipeng Shao, Lubomir Sokol, Eduardo M Sotomayor, Ling Zhang

**Affiliations:** Department of Pathology, Chinese Academy of Medical Science, Peking Union Medical College Hospital, Beijing, China; Department of Anatomic Pathology, H. Lee Moffitt Cancer Center and Research Institute, Tampa, FL USA; Department of Sarcoma, H. Lee Moffitt Cancer Center and Research Institute, Tampa, FL USA; Department of Hematopathology and Laboratory Medicine, H. Lee Moffitt Cancer Center and Research Institute, Tampa, FL USA; Department of Malignant Hematology, H. Lee Moffitt Cancer Center and Research Institute, Tampa, FL USA

**Keywords:** Primary bone lymphoma (PBL), Secondary bone lymphoma (SBL), Diffuse large B-cell lymphoma (DLBCL), Clinico-pathological characteristics, Prognostic factors, Multifocal bone involvement/multifocality

## Abstract

**Background:**

Malignant bone lymphoma can be classified as primary (PBL) or secondary (SBL) bone lymphoma. However, the clinico-pathological characteristics and prognostic factors of PBL versus SBL have not yet been well defined. Whether lymphoma with multifocal bone involvement should be considered as stage IV PBL or SBL still remain controversial throughout the literature.

**Methods:**

In this study, we retrospectively reviewed 127 patients with bone lymphoma diagnosed from1998 to 2013 at the Moffitt Cancer Center. Patients were classified as PBL (81 cases) and SBL (46 cases) using the 2013 WHO Classification of Bone/Soft Tissue Tumors and PBL patients were further subdivided into: 1) PBL with unifocal bone disease (uPBL, 46 cases), 2) PBL with multifocal bone involvement (mPBL, 35 cases). Patient characteristics, survival, and prognostic factors were analyzed.

**Results:**

Diffuse large B-cell lymphoma (DLBCL) was the most common histological subtype in all three groups (37/46 of uPBL, 23/35 of mPBL, 23/46 of SBL). B symptoms, lymph node involvement, and bone marrow involvement were found to be more common in mPB-DLBCL and SB-DLBCL groups than in the uPB-DLBCL group. Femur was found to be the most common affected site in uPB-DLBCL patients, while spine was most commonly involved in the other two groups. Survival analysis indicated that uPBL-DLBCL patients had a significantly better progression-free survival (PFS) and overall survival (OS) than those in the other two groups (*P* < 0.05). We also found by univariate analysis that multifocality, and stage IV were significantly poor prognostic factors for both PFS and OS in PBL patients. Using multivariate analysis, multifocality remained an independent prognostic factor for both PFS and OS (*P* = 0.0117, RR: 3.789, 95% CI: 1.275-11.256).

**Conclusion:**

Overall, our results suggest that mPBL is more similar to SBL in characteristics and survival rather than uPBL, and thus should be better classified and treated as SBL.

**Electronic supplementary material:**

The online version of this article (doi:10.1186/1471-2407-14-900) contains supplementary material, which is available to authorized users.

## Background

Malignant bone lymphomas are uncommonly encountered clinically. According to the initial extent of disease, malignant bone lymphomas can be divided into two groups: primary bone lymphoma (PBL) and secondary bone lymphoma (SBL) [1, 2]. PBL is an extremely rare entity, accounting for approximately 7% of malignant bone tumors, 5% of extra-nodal lymphomas, and <1% of all non-Hodgkin lymphomas [1, 3]. It has been considered to have the best prognosis of all primary malignant bone lesions. However, SBL is more common, seen in approximately 16% to 20% of patients with lymphoma, and has a relatively poor prognosis [1]. Given their different clinical outcomes and treatment strategies, subclassification of bone lymphomas into either primary or secondary bone lymphomas is critical. A review of the literature shows that there is no consensus regarding how to accurately distinguish PBL from SBL. The most common debate falls in how to subclassify and treat bone lymphoma when it primarily presents with multifocal bone disease with/without regional lymph node and/or adjacent soft tissue involvement. The reported 5-year overall survival (OS) rates vary between different study groups of PBL patients due to different diagnostic criteria, ranging from less than 36% to more than 88.3% [4–8]. In addition, the clinico-pathological characteristics and prognostic factors of PBL versus SBL have not yet been well studied.

Recently, the 2013 World Health Organization (WHO) classification of bone/soft tissue tumors [1] defined PBL as a neoplasm composed of malignant lymphoid cells, producing one or more masses within bone, without any supra-regional lymph-node involvement or other extra-nodal lesions. According to the criteria, bone lymphoma with/without regional lymph node and without other extra-nodal lesions was also classified as PBL clinically, regardless of whether the bone lesion occurred unifocally or multifocally. We recently identified several potential prognostic factors using the new 2013 WHO classification in a large study-cohort including 70 PBL cases and showed that soft tissue extension and IPI score were the most important unfavorable prognostic indicators for both PFS and OS in PBL [9]. However, limited information was available per literatures on the prognostic role of multifocality in PBL, and whether PBL with multifocal bone involvement should be considered as SBL. Here, we conducted a single-center retrospective study in which we classified bone lymphoma as PBL and SBL using the new 2013 WHO classification of soft tissue neoplasms, further subcategorized PBL patients into two groups, those with unifocal bone disease (uPBL) and those with multifocal bone involvement (≥2 foci) (mPBL), and compared patient characteristics, treatments and outcome among uPBL, mPBL and SBL groups, aiming to further explore the clinical and prognostic significance of multifocal bone involvement in PBL and to clarify the current definition of PBL.

## Methods

### Patients

Chart records of 145 patients with biopsy-proven malignant bone lymphoma were retrieved from the surgical pathology files of Moffitt Cancer Center diagnosed over a 15-year-period (1998–2013), following the guidelines of the Moffitt Cancer Center Scientific Research Committee and with the approval of the Institutional Review Board at the University of South Florida. After an initial review of the clinical and pathological data, 18 patient records were excluded because of the inadequacy of staging and/or follow-up information. Patient characteristics, survival, and prognostic factors were analyzed. Medical records were reviewed for age, sex, race, involved sites, lactate dehydrogenase (LDH), pathological diagnosis, treatments, date of diagnosis, lymph node involvement, bone marrow involvement, stage and date of disease progression, relapse, death, or last follow-up. Lymph node involvement was demonstrated by a clinical and imaging enlargement of node (>1.5 cm measured per the Positron Emission Tomography, PET scan) with or without an excision biopsy. Our study thus included 127 patients with bone lymphomas. We classified patients as PBL and SBL using the updated 2013 WHO criteria for bone/soft tissue tumors [1] and then further subcategorized PBL into two subgroups:1) uPBL(n = 46),2) mPBL(n = 35). Bone marrow involvement was assessed by an aspiration and bone marrow biopsy from iliac crest. If the primary lesion is near iliac or pelvic region, contra-lateral ilic crest is used for the biopsy site. Bone lymphoma with distant bone marrow involvement as the only other site of extranodal disease was also classified as PBL (stage IV) in our study, because a number of previous studies have demonstrated that it has a similar prognosis to PBL with localized disease [2, 7, 10]. Given the relatively rarity of the other histological subtype, only patients with diffuse large B-cell lymphoma (DLBCL), were further reviewed for patient characteristics and analyzed for prognostic factors in the current study.

### Histological diagnosis and immunohistochemistry findings

All patients were diagnosed lymphoma by bone biopsy. Morphologic assessments in conjunction with flow cytometry (only if fresh tissue had been harvested) or immunohistochemical (IHC) study were conducted. The IHC markers included CD20, PAX-5, CD10, Bcl-2, Bcl-6, or MUM-1 for DLBCL or large B-cell lymphoma, unclassifiable, with features between DLBCL and Burkitt lymphoma (BLUI) and CD30, CD3, CD4, CD8, CD43, granzyme B and anaplastic lymphoma kinase (ALK) for anaplastic large T-cell lymphoma (ALCL).

### Staging

Patients were staged retrospectively according to the Ann Arbor staging system as described before [9]. In all cases, staging evaluation included *1*) a chest X-ray or a computed tomography (CT) scan of the chest, *2*) a CT scan or ultrasonogram of the abdomen and pelvis, 3) whole body bone scan or positron emission tomography–computed tomography (PET-CT) scan or magnetic resonance imaging (MRI), and 4) bone marrow biopsy of iliac bone.

### Survival analysis

Progression-free survival (PFS) was defined as the interval from the date of diagnosis to the date of disease progression, relapse, or death from any cause. Patients who showed no progression were censored at the date of most recent available radiographic imaging. OS was calculated from the date of diagnosis to the date of death from any cause using the Social Security Death Index (SSDI). For unknown deaths, patients were censored at last follow-up. Survival curves were calculated according to the Kaplan-Meier method and compared using the log-rank test. Differences were considered significant if *P* values were ≤0.05 (two-tailed). Multivariate analysis was performed using a Cox model using a forward variable selection procedure. Only the variables with significant values (P ≤ 0.05) in univariate analysis were included in the multivariate analysis. All data analyses were performed by SPSS software for windows, version 20 (SPSS Inc., Chicago, IL).

## Results

### Histological diagnosis and patient characteristics

The histological classification of our series is shown in Table 1. DLBCL was the most common histological subtype in all three groups. However, the proportion of DLBCL patients in the SBL group was significantly lower than that in the uPBL group (23/46, 50% versus 60/71, 85.7%) (*P* < 0.05). Classical Hodgkin lymphoma and follicular lymphoma were more commonly shown in SBL group, while only 1 classical Hodgkin lymphoma case was identified in the PBL groups. T-cell lymphoma is relatively rare, with four of the total six T-cell lymphoma cases in the mPBL group. All classical Hodgkin lymphoma cases had nodular sclerosis histology. Among the 127 bone lymphoma patients, only two PBL cases were HIV positive, including one DLBCL and one large B-cell lymphoma, unclassifiable, with features intermediate between DLBCL and Burkitt lymphoma (BLUI).

**Table 1:** **Histopathological subtypes of patients with bone lymphoma**

Classification	uPBL	mPBL	SBL
DLBCL, n (%)	37(80.4)	23(65.7)	23(50.0)
Follicular lymphoma, n (%)	4(8.7)	3(8.6)	9(19.6)
Small lymphocytic lymphoma, n (%)	1(2.2)	1(2.9)	0(0)
Marginal zone lymphoma, n (%)	1(2.2)	1(2.9)	2(4.3)
Not further subclassified^a^, n (%)	1(2.2)	2(5.7)	1(2.2)
BLUI , n (%)	0	1(2.9)	0(0)
Classical Hodgkin lymphoma, n (%)	1(2.2)	0(0)	10(21.7)
T cell, n (%)	1(2.2)^b^	4(11.4)^c^	1(2.2)^d^
Total, n (%)	46	35	46

Abbreviations uPBL: primary bone lymphoma with unifocal bone disease; mPBL: primary bone lymphoma with multifocal bone disease; SBL: secondary bone lymphoma; DLBCL: diffuse large B-cell lymphoma; Large B-cell lymphoma, unclassifiable, with features intermediate between DLBCL and Burkitt lymhoma: BLUI; ALCL: anaplastic large T-cell lymphoma; PTCL, NOS: peripheral T-cell lymphoma, not otherwise specified.

^a^Low-grade, small B-cell lymphoma no further information for subclassification.

^b^ALCL(n = 1).

^c^ALCL (n = 2), T-lymphoblastic lymphoma (n = 1), and PTCL, NOS (n = 1).

^d^ALCL (n = 1).

Given the histological heterogeneity and the relative rarity of the other histological types, only DLBCL patients were further explored for demographical and clinical characteristics as well as survival. The characteristics of the 83 DLBCL patients are summarized in Tables 2 and 3. Compared with primary bone DLBCL with unifocal bone disease (uPB-DLBCL), B symptoms, lymph node involvement, and bone marrow involvement were more commonly shown in the other two groups: primary bone DLBCL with multifocal bone disease (mPB-DLBCL) and secondary bone DLBCL (SB-DLBCL). No significant differences regarding age distribution were shown among three groups.

**Table 2:** **Patient demographics and clinical characteristics of bone DLBCL**

Parameter	uPB-DLBCL	mPB-DLBCL	SB-DLBCL
**Total**	37	23	23
**Age range, years**	15-89	17-82	29-82
Median age, years	53.0	60.0	54.0
Mean age, years	52.9	53.4	55.0
<60, n (%)	22(59.5)	11(47.8)	15(65.2)
≥60, n (%)	15(40.5)	12(52.2)	8(34.8)
**Sex**			
Male, n (%)	22(59.5)	14(60.9)	9(39.1)
Female, n (%)	15(40.5)	9(39.1)	14(60.9)
Male :female ratio	1.5:1	1.6:1	0.6:1
**Race**			
White, n (%)	33(89.2)	21(91.3)	17(73.9)
Black, n (%)	2(5.4)	1(4.3)	3(13.0)
Unknown, n (%)	2(5.4)	1(4.3)	3(13.0)
**B symptoms, n (%)**	7(18.9)	8(34.8)	8(34.8)
**LDH**			
Normal	23(79.3)	5(29.4)	8(57.1)
Elevated	6(20.7)	12(70.6)	6(42.9)
**Lymph node involvement, n (%)**	4(10.8)*	9(39.1)*	14(60.9)**
**Bone marrow involvement, n (%)**	3(8.1)	5(21.7)	7(30.4)
**Number of bone sites, n (%)**			
Unifocal	37(100.0)	0(0)	7(30.4)
Multifocal	0(0)	23(100.0)	16(69.6)
**Sites, n (%)**			
Appendicular	29(78.4)	10(43.5)	7(30.4)
Axial	8(21.6)	2(8.7)	9(39.1)
Both	0(0)	11(47.8)	7(30.4)
**Stage, n (%)**			
IE	30(81.1)	0(0)	1(4.3)
IIE	4(10.8)	0(0)	2(8.7)
IIIE	0(0)	0(0)	0(0)
IVE	3(8.1)	23(100.0)	20(87.0)

*Abbreviations* uPB-DLBCL: primary bone diffuse large B-cell lymphoma with unifocal bone disease; mPB-DLBCL: primary bone diffuse large B-cell lymphoma with multifocal bone disease; SB-DLBCL: secondary bone diffuse large B-cell lymphoma.

*Patients with regional lymph node enlargement.

**Patients with both regional and supraregional or systemic lymph node involvements.

**Table 3:** **Common involved sites of bone DLBCL**

Sites	uPB-DLBCL (N = 37)	mPB-DLBCL (N = 23)	SB-DLBCL (N = 23)
**Extremities, n (%)**			
Femur	12(32.4)	12(52.2)	7(30.4)
Humerus	6(16.2)	5(21.7)	3(13.0)
Tibia	5(13.5)	6(26.1)	2 (8.7)
**Pelvis, n (%)**	5(13.5)	13(56.5)	9 (39.1)
**Spine, n (%)**	7(18.9)	13(56.5)	11 (47.8)

*Abbreviations* uPB-DLBCL: primary bone diffuse large B-cell lymphoma with unifocal bone disease; mPB-DLBCL: primary bone diffuse large B-cell lymphoma with multifocal bone disease; SB-DLBCL: secondary bone diffuse large B-cell lymphoma.

Femur was most commonly involved in the uPB-DLBCL group. However, spine was the most common affected site in the other two groups. Pelvis, humerus, and tibia were also commonly involved in our series.

Most patients in the uPB-DLBCL group were classified as stage IE (unifocal localized bone lesions without lymph node involvement). In the mPB-DLBCL group, all patients were staged as IVE on the basis of multifocal bone involvement. The majority of SB-DLBCL patients were classified as stage II-IVE. Only 1 patient with SB-DLBCL, who had presented with unifocal bone disease and without lymph node or other extra-nodal sites involvement when disease relapsed, was classified as stage I.

### IHC findings

IHC study was performed in only a subset of patients with PB-DLBCL. The available data are summarized as follows: approximately half (26/43) were CD10-positive. Bcl-2, Bcl-6, and MUM-1 expression were detected in 19 of 23 (82.6%), 24 of 27 (88.9%), and 2 of 16 (12.5%) patients, respectively. In situ hybridization using Epstein-Barr virus-encoded RNA probe was performed in five PB-DLBCL cases, with all five being negative.

Besides CD30, CD3, CD4, CD8, CD43, and granzyme B, ALK IHC staining was performed in all three primary bone ALCL cases, with two of the three being positive.

### Treatments

Treatments of DLBCL patients were summarized (Table 4). Most patients with uPB-DLBCL with received combined modality therapy (chemotherapy and radiotherapy), whereas more than half of SB-DLBCL patients with bone involvement at presentation and mPB-DLBCL patients were treated with chemotherapy alone. Most bone DLBCL patients received CHOP or CHOP-like chemotherapy with rituximab, and only eight DLBCL patients received CHOP or CHOP-like chemotherapy alone without rituximab. R-ESHAP (rituximab plus etoposide, methylprednisolone, cytarabine, cisplatin) was the main salvage therapy for SB-DLBCL with recurrent bone involvement.

**Table 4:** **Treatments of bone DLBCL**

Parameter	Number (%)		
	uPB-DLBCL	mPB-DLBCL	SB-DLBCL*
**Treatment**	n = 37	n = 23	n = 12
CMT	11(29.7)	16(69.6)	7(58.3)
RT	1(2.7)	0	0
CMT and RT	24(54.1)	6(26.1)	5(41.7)
No CMT or RT	1(2.7)	1(4.3)	0
**Chemotherapy**	n = 35	n = 22	n = 12
CHOP or CHOP-like CHOP-like	7(20.0)	0	1(8.3%)
CHOP + rituximab	28(80.0)	22(100.0)	11(91.7%)

*Abbreviations* uPB-DLBCL: primary bone diffuse large B-cell lymphoma with unifocal bone disease; mPB-DLBCL: primary bone diffuse large B-cell lymphoma with multifocal bone disease; SB-DLBCL: secondary bone diffuse large B-cell lymphoma; CMT: chemotherapy; RT: radiation therapy.

*SB-DLBCL with recurrent bone involvement was not included in the table.

### Survival analysis of patients with bone lymphoma

**Figure 1:**
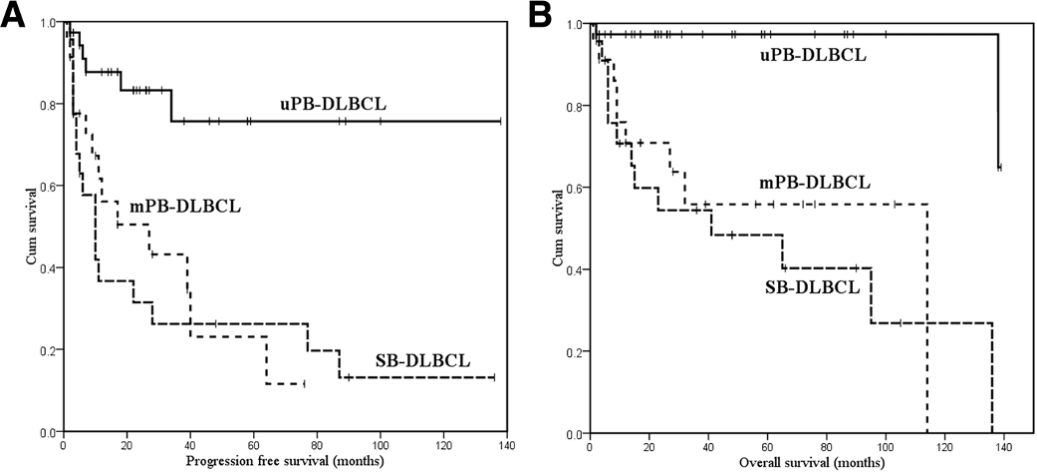
**Overall survival (A) and progression-free survival (B) in three groups of bone DLBCL.**

Patient follow-up time was calculated using reverse Kaplan-Meier analysis. For 83 bone DLBCL patients, the median follow-up times for PFS and OS were 28 months (range, 1–138 months) and 38 months (range, 1–139 months), respectively. PFS and OS data for uPB-DLBCL, mPB-DLBCL and SB-DLBCL groups are illustrated in Figure 1. The 5-year PFS rates were 75.7% for uPB-DLBCL, 13.4% for mPB-DLBCL, and 22.0% for SB-DLBCL (Figure 1A). The 5-year OS rates were 83.4% for uPB-DLBCL, 36.7% for mPB-DLBCL and 41.9% for SB-DLBCL (Figure 1B). uPBL patients had a significantly better PFS and OS than those in the other two groups (PFS: *P* = 0.001 for uPB-DLBCL vs. mPB-DLBCL, *P* < 0.001 for uPB-DLBCL vs. SB-DLBCL; OS: *P* < 0.001 for uPB-DLBCL vs. mPB-DLBCL, *P* < 0.001 for uPB-DLBCL vs. SB-DLBCL). There were no significant differences in either PFS or OS between the other two groups (PFS: *P* = 0.732; OS: *P* = 0.572).

Similar results were obtained for our total series of 127 bone lymphoma (Additional file 1: Figure S1).

### Prognostic factor analyses

We analyzed the influence of the following individual factors on survival in PB-DLBCL patients: age, sex, B symptoms, LDH, lymph node involvement, bone marrow involvement, involved sites, the number of bone sites, stage, and IHC markers (CD10, Bcl-2, Bcl-6, and MUM-1). In univariate analysis, LDH, involvement of both appendicular and axial sites multifocality, and stage IV were significant poor prognostic factors for both PFS and OS (Table 5). Age ≥ 60 years was also a significant poor prognostic factor for OS (Table 5). None of the IHC markers were significant predictors for PFS or OS. Using Cox regression for multivariate analysis, multifocality were independent unfavorable prognostic factors for both PFS and OS (Table 6). Age ≥ 60 years was again an independent unfavorable prognostic factor for OS.

**Table 5:** **Univariate analysis of prognostic factors for survival in patients with PB-DLBCL**

Parameter	PFS			OS		
	3-year	5-year	***P***	3-year	5-year	***P***
**Overall, %**	60.8	45.9		78.8	68.0	
**Age**			0.278			**0.004**
<60 years, %	64.5	46.9		87.9	80.6	
≥60 years, %	50.3	40.2		57.9	44.3	
**Sex**			0.634			0.410
Male, n (%)	63.9	52.1		80.2	71.0	
Female, n (%)	53.4	44.8		71.9	61.4	
**B symptoms**			0.673			0.289
No, %	62.5	52.4		80.5	74.3	
Yes, %	50.0	31.5		66.1	45.8	
**LDH**			**0.011**			**0.018**
Normal, %	73.6	58.2		89.2	68.2	
Elevated, %	34.8	21.9		55.2	38.2	
**Lymph node involvement**			**0.143**			0.231
No, %	66.8	60.0		81.4	75.1	
Yes, %	15.6	2.2		64.0	44.3	
**Bone marrow involvement**			0.719			0.319
No, %	59.1	42.0		80.0	72.3	
Yes, %	72.9	72.9		54.9	42.0	
**Sites**			**0.022**			**0.022**
Appendicular, %	66.7	51.1		78.4	70.6	
Axial, %	59.1	59.1		100.0	100.0	
Both, %	5.6	0		61.4	61.4	
**Number of bone sites**			**0.001**			**<0.001**
Unifocal, %	75.7	75.7		89.2	83.5	
Multifocal, %	36.8	13.4		53.1	36.7	
**Stage**			**0.005**			**0.001**
IE/IIE, %	73.8	73.8		97.1	97.1	
IVE, %	43.0	18.0		58.5	49.8	
**CD10**			0.267			0.077
Negative, %	52.9	29.8		76.8	65.2	
Positive, %	64.4	55.2		95.7	95.7	

Bold values indicate statistical significance (P<0.05).

**Table 6:** **Multivariate analysis of prognostic factors for patients with PB-DLBCL**

Parameter	PFS			OS		
	RR	95%CI	***P***	RR	95%CI	***P***
**Number of bone sites**			**0.015**			**0.014**
Unifocal	Reference group			Reference group		
Multifocal	3.728	1.292-10.754		20.061	1.851-217.377	
**Age**						**0.035**
<60 years				Reference group		
≥60 years				15.791	1.215-205.200	

Bold values indicate statistical significance (P<0.05).

Moreover, as for SBL, we found that all three patients with recurrent lymphoma presenting with unifocal bone disease as the only involved site (re-stage I) (one DLBCL, one low-grade B cell lymphoma, and one classical Hodgkin lymphoma) survived without disease progression until final follow-up (3, 104, and 136 months, respectively).

## Discussion

PBL was first described by Oberling in 1928 [11] and is thought to be a separate disease entity from conventional nodal or extranodal base lymphoma with an excellent prognosis. Up to now, its definition still remains controversial, especially regarding whether multifocal bone involvement by lymphoma at initial presentation without any supra-regional lymph node involvement and other extra-nodal disease should be defined as PBL [10]. Of importance, with obvious improvements in imaging technology in recent decades, the proportion of patients diagnosed with multifocal bone lymphoma has increased [7, 12]. Thus, these discrepancies in PBL definition and the improvements in diagnostic procedures have led to difficulties in the comparison of clinic-pathological characteristics and clinical outcomes between studies. In addition, it also raises the question regarding whether multifocality of bone lymphoma should be considered as an independent prognostic predictor. Although there have been several studies on malignant bone lymphoma, these have thus far been limited by small sample sizes and/or have included only early-stage PBL cases [4, 13, 14]. Here, we describe a relatively large cohort of PBL patients (n = 81) and a compared group of SBL patients (n = 46) diagnosed and treated during 1998–2013 at our institution with modern and contemporary diagnostic and therapeutic modalities. A relatively high proportion of our PBL patients (43.2%, 35 of 81 cases) presented with multifocal bone disease. This may be due to the routine use of PET, CT, MRI, and bone scanning for staging. Because only bone biopsy-proven cases were selected, the number of SBL patients was relatively small in our series.

Our initial analysis of patient characteristics (age and sex distribution) was consistent with previous studies [10, 15]. However, mPB-DLBCL and SB-DLBCL patients had higher frequency of B symptoms, lymph node involvement, and iliac bone marrow involvement than uPB-DLBCL patients. In previous studies, femur has been reported to be the most commonly involved site in PBL [3, 16, 17]. In our series, femur was also found to be the most common affected site in uPB-DLBCL patients. However, spine was most commonly involved in mPB-DLBCL and SB-DLBCL. From this point of view, our results suggest that mPBL is more similar to clinical characteristics of patients with SBL rather than with uPBL.

Consistent with previous studies, DLBCL was the most common histological subtype in our bone lymphoma series. However, the uPBL group had a significantly higher proportion of DLBCL (80.4%, 37 of 46 cases) than the SBL group (50.0%, 23 of 46 cases). Our results indicate that the histological distributions are different between the uPBL group and the SBL group.

Regarding the subclassification of PBL, several studies with small sample sizes have described the IHC characteristics of PB-DLBCL [18–21]. In these previous reports, approximately half of the PB-DLBCL cases demonstrated a germinal center B-cell (GCB) phenotype by IHC with high Bcl-2 and/or Bcl-6 expression and relatively low MUM-1 expression. We also observed high percentages of Bcl-2 and Bcl-6 expression in our series. However, incomplete IHC data of MUM-1 in our study precluded an accurate subclassification of our PB-DLBCL cases into GCB or non-GCB subgroups. Despite so, 26 of 43 patients were able to be classified with PB-DLBCL in our series according to CD10-positivity, which meant that at least 60.5% of these patients were of GCB phenotype. Prior studies have yielded conflicting results about the predictive value of these IHC markers, particularly of CD10 and GCB stubtype [5, 18–21]. Although insufficient for subclassification of GCB or non-GCB subtype of PB-DLBCL, our limited data showed no association between various markers (CD10, Bcl-6, Bcl-2, MUM-1) and survival in PB-DLBCL.

In the study, we temporally subgrouped the patients with mPBL as stage IV since whether lymphoma with multifocal bone involvement should be considered as stage IV PBL or SBL still remain controversial in the literature. Because of the unequivocal definition of PBL, some previous studies restricted diagnoses to those with early-stage PBL (stage IE and IIE) [14, 22]. Only a few studies have focused on the significance of multifocal bone diseases in PBL [10, 15]. In our study, patients classified with uPB-DBLCL had an excellent prognosis, whereas those with mPB-DLBCL carried a poor prognosis, with survival being similar to SB-DLBCL. The finding suggests that those with mPBL would benefit from being classified as SBL rather than conventional PBL. Further prognostic factor analyses also revealed that multifocality was an independent prognostic factor of PB-DLBCL, which also supports that mPBL may be a different clinical entity from uPBL. Although unifocal bone lymphoma, in general, can be eradicated with local radiation in 50% of patients, the treatment of patients with multifocal osseous disease, especially those presenting with associated soft tissue invasion or generalized adenopathy, is much less satisfactory [23]. The treatment modality was also somewhat different among PB-DLBCL and SB-DLBCL groups in our study. Most patients with uPB-DLBCL were treated with combined modality therapy (chemotherapy and radiotherapy) for localized lesions, whereas mPB-DLBCL and SB-DLBCL typically received chemotherapy alone. Given that mPBL and SBL patients had similar clinical characteristics, prognosis, and treatment modality, our data suggest that it would be better to classify so-called “mPBL” as SBL in particular under the setting of DLBCL. As known, DLBCL constitutes the majority of PBL. Thus, we consider that the current definition for PBL might need further clarification. In clinically and radiologically advanced-stage PBL patients having multiple bone site involvement, especially in those with regional lymph node and/or adjacent soft tissue involvement, it may be impossible to distinguish mPBL from SBL. According to our results, it might not be necessary to distinguish mPBL from SBL clinically.

In a study by Jawad et al. [10], it was suggested that the use of the name “PBL” should be limited to those with truly local disease with a single osseous lesion. This is also the reason we limited mPBL to those with stage IV in the study. Although stage IV itself was also a significant poor prognostic factor for survival in PBL patients by univariate analysis, it failed to show independent prognostic significance in multivariate analysis, probably due to the strong correlation between stage IV and multifocal bone involvement. Ostrowski et al. [15] also reported that those with malignant lymphoma with multifocal bone disease had a significantly poorer survival than those with unifocal bone involvement. However, their study demonstrated that prognosis of patients with malignant lymphoma with multifocal bone disease was considerably better than those having SBL. Two main reasons may explain the difference from our study. First, their SBL group included a high proportion of patients with malignant lymphoma with recurrent bone involvement when compared with our data. Second, their patients with regional lymph node involvement and/or soft tissue extension were excluded from the group of multifocal bone involvement.

Furthermore, due to the rarity of PBL patients who present with regional lymph node and/or bone marrow involvement, there is no consensus regarding the effects of regional lymph node or bone marrow involvement on survival in patients with PBL. No significant association was observed between regional lymph node or bone marrow involvement and survival in our PB-DLBCL patients, suggesting that it was reasonable to categorize these cases into PBL rather than SBL with a relatively worse prognosis. However, our results should be interpreted with caution given the small sample size.

As for SBL, although recurrent lymphoma is usually associated with a poor prognosis, we found that patients with recurrent lymphoma presenting with unifocal bone disease as the only involved site (re-stage I) had an excellent prognosis. This result needs careful interpretation, taking into consideration that our study included only 3 patients with stage I SBL.

It has been mentioned that low grade B-cell lymphoma, T-cell lymphoma and Hodgkin lymphoma have also been included in the study. As the minority in PBL, we are unable to perform risk stratification for these lymphomas. Multicenter studies with larger number of cases are warranted to explore their prognostic values in PBL. However, it is of worthy for us to learn several interesting findings in the study. Our series also confirms that primary bone Hodgkin lymphoma is extremely rare (1 of 81 PBL patients) in contrast to secondary bone Hodgkin lymphoma (10 of 46 patients, 21.8%) as reported in literatures, 10-20% [3, 17]. Only five primary T-cell lymphoma cases (including 3 ALCLs) were included in our PBL series. All five cases showed rapid disease progression within the first year after diagnosis, with three deceased 4–8 month after diagnosis (data not shown). Consistent with our results, in the study by Hsieh et al. [5], all five patients with primary bone T-cell lymphoma (including 4 ALCLs) with follow-up information died within 1 year. Limited case number precludes a further prognostic analysis. Similarity also applies to primary bone Hodgkin lymphoma. Given the small case number and histological heterogeneity in low grade B-cell lymphomas, no further studies have been conducted in our study, either.

## Conclusions

In summary, our study retrospectively described our single institution experience with 127 bone lymphoma patients, including 81 cases of PBL and 46 cases of SBL using the new 2013 WHO criteria. Patients with mPB-DLBCL and SB-DLBCL showed similar characteristics, with both having a poorer outcome, whereas uPB-DLBCL patients demonstrated somewhat different characteristics and had an excellent outcome. Moreover, multifocality was found to be an independent prognostic factor of PB-DLBCL. Due to the similar patient characteristics and outcome, it would be better to classify bone lymphoma presenting with multifocal bone disease as SBL rather than conventional PBL, regardless of whether there is supraregional lymph node or other extranodal site involvement. Our results indicate that the current criteria for PBL need further clarification, and it might be unnecessary to distinguish mPBL from SBL, clinically. Given the relatively small sample size of patients with SBL and the incomplete IHC data, our results warrant further clarification in large multicenter studies.

## Electronic supplementary material

Additional file 1: Figure S1: Overall survival (A) and progression-free survival (B) in three groups of bone lymphoma (OS: *P* = 0.034 for uPBL vs. mPBL, *P* < 0.001 for uPBL vs. SBL, *P* = 0.074 for mPB vs. SBL; PFS: *P* = 0.347 for uPBL vs. mPBL, *P* < 0.001for uPBL vs. SBL, *P* = 0.517for mPB vs. SBL). (TIFF 133 KB)
