# Supplementary figures and images for: Clinical characteristics and prognostic factors of bone lymphomas: focus on the clinical significance of multifocal bone involvement by primary bone large B-cell lymphomas
Source: BMC Cancer. 2014 Dec 2;14:900. doi: 10.1186/1471-2407-14-900 (PMC4265495; doi:10.1186/1471-2407-14-900)

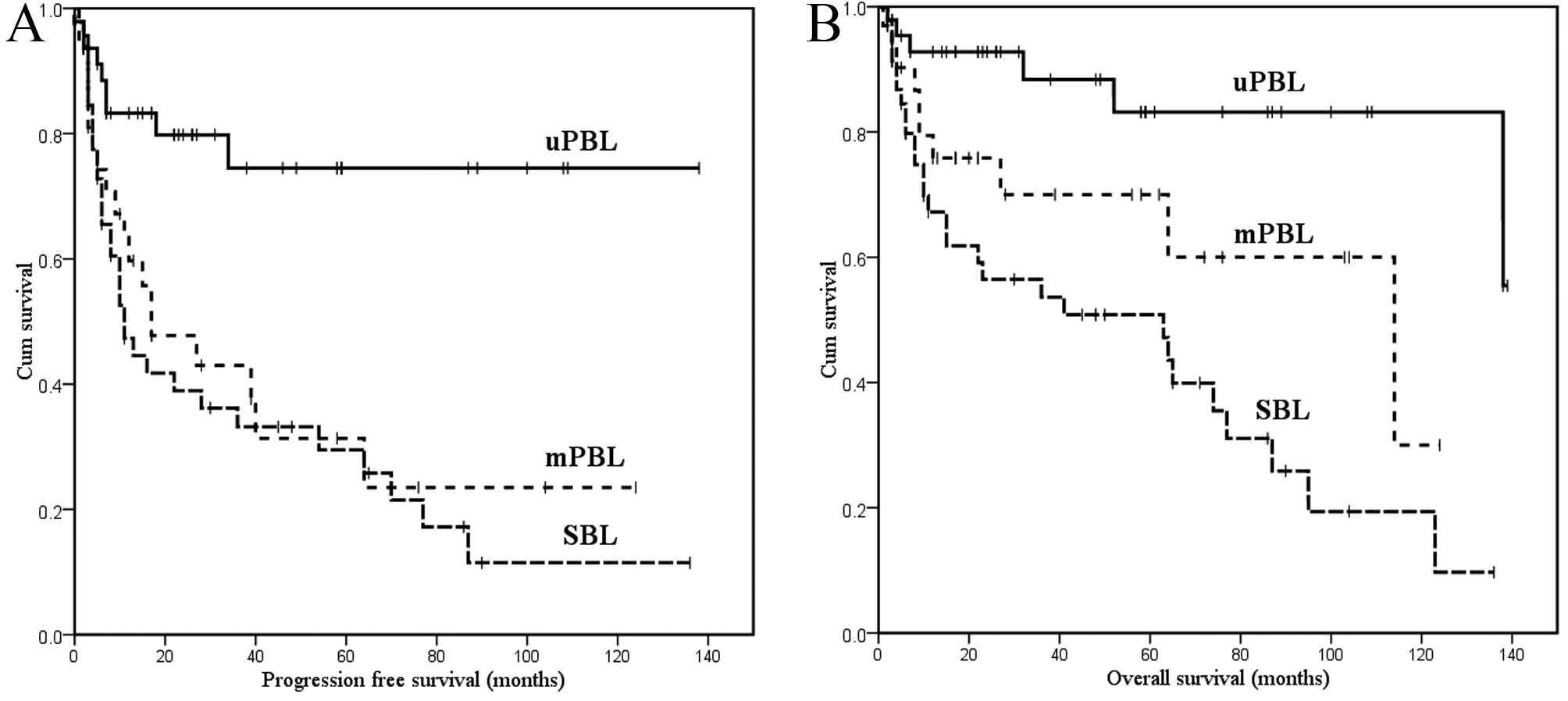

Supplement: Supplementary file 1 — Additional file 1: Figure S1: Overall survival (A) and progression-free survival (B) in three groups of bone lymphoma (OS: P = 0.034 for uPBL vs. mPBL, P < 0.001 for uPBL vs. SBL, P = 0.074 for mPB vs. SBL; PFS: P = 0.347 for uPBL vs. mPBL, P < 0.001for uPBL vs. SBL, P = 0.517for mPB vs. SBL). (TIFF 133 KB) [file 12885_2014_5067_MOESM1_ESM.tiff]
